# Cytokine profile in elderly patients with sepsis

**DOI:** 10.4103/0972-5229.56052

**Published:** 2009

**Authors:** Anil T. Kumar, U. Sudhir, K. Punith, Rahul Kumar, V. N. Ravi Kumar, Medha Y. Rao

**Affiliations:** **From: ** Department of Medicine, M S Ramaiah Medical Teaching Hospital, MSRIT Post, New BEL Road, Bangalore - 560 054, India

**Keywords:** Cytokine profile, IL-6, prognosis, sepsis, TNF-α

## Abstract

**Context:**

Sepsis is a serious health problem in the elderly with a high degree of mortality. There is very limited data available in elderly subjects regarding the markers for sepsis. Development of good markers will help in overall management and prediction of sepsis.

**Objectives:**

Serial estimation of Interleukin-6 (IL-6) and Tumor Necrosis Factor-Alpha (TNF-α) and their correlation with mortality in sepsis in elderly patients and to determine the influence of gender on cytokine production and mortality in elderly patients with sepsis.

**Settings and Design:**

The prospective study was conducted at our tertiary care center from April 2007 to September 2008. Elderly Patients satisfying the Systemic Inflammatory Response Syndrome (SIRS) criteria were included.

**Methods and Material:**

TNF-α and IL-6 were estimated in 30 elderly patients admitted to our intensive care unit with SIRS and sepsis. The estimations were done on day 1, 3 and 7 of admission.

**Statistical Analysis Used:**

Student and paired ‘t’ tests, and ANOVA, which were further followed up by post-hoc ‘t’ tests with Bonferroni correction using SPSS.

**Results:**

Reducing levels of IL-6 levels from day 1 to 7 was found in the survivor group. TNF-α level was significantly low on day 1 in the nonsurvivor female group.

**Conclusions:**

Serial estimation of cytokines in elderly patients with sepsis will help in prediction of mortality. Female gender was an independent predictor of increased morality in critically ill patients with sepsis.

## Introduction

Sepsis is defined as the systemic host response to infection.[1] Despite rapid progress in health care over the past decades, sepsis continues to be a major life threatening condition in acute care patients, especially in the age group above 60 years.[2] It is the most common cause of death in hospitalized patients, affects over 18 million people worldwide and has an expected 1% increase in incidence per year.[3] Indian data also revealed high admission rates and mortality in patients admitted to intensive care units.[4] Presently, the diagnosis and management of patients with sepsis is based on clinical and laboratory data with poor accuracy.[5] Given this substantial mortality and economic costs, a basic understanding of pathogenesis and the immune alterations in sepsis may help to direct therapy.

The study of pathophysiology of sepsis has lead to the development of markers. Markers help in establishing diagnosis, quantifying the severity and assessing the response to therapy. There are about 80 markers described in sepsis.[6] Tumor Necrosis Factor-Alpha (TNF-α) is by far the most well studied pro-inflammatory cytokine studied in sepsis. Interleukin-6 (IL-6) is a pleotropic cytokine with potent stimulatory effect on macrophages and B and T lymphocytes. Among the milieu of cytokines induced during sepsis, the best correlation of plasma cytokine concentration with mortality rate has been made with IL-6.[7,8]

Since very limited data is available in elderly subjects regarding the markers for sepsis, the following study was undertaken to establish the cytokine profile in sepsis in the elderly and develop markers for prediction.

A serial estimation of IL-6 and TNF-α and their correlation with mortality in sepsis were done in elderly patients. The influence of gender on cytokine production and mortality in elderly patients with sepsis was considered in particular in the present study.

## Materials and Methods

The prospective study was conducted at our tertiary care center from April 2007 to September 2008. The study was approved by the institution's ethics committee. We included 30 consecutive patients admitted with strong suspicion of infection to the medical wards at the study center. Patients were eligible for inclusion if they were aged above 60 years satisfying the Systemic Inflammatory Response Syndrome (SIRS) or organ dysfunction criteria.[1] Patients with presence of leukocytes in normal sterile body fluid or perforated viscous or radiographic evidence of pneumonia with purulent sputum or any syndrome associated with high risk of infection were also included in the study. Patients with HIV infection, pre-existing malignancies, autoimmune diseases, post transplant status, known or suspected chronic liver disease, acute pancreatitis and chronic renal failure were excluded from the study. An array of investigations were done on admission to check for the inclusion criteria satisfaction (Complete hemogram, blood grouping and Rh-typing, bleeding time, clotting time, activated partial thrombin time, fibrin degradation products, HIV ELISA, urine examination, sputum examination, stool examination, analysis of body fluids, renal function tests, liver function tests, chest x-ray, arterial blood gas analysis, APACHE-II scoring and cultures of endotracheal tube suction, catheter, IV line and cerebrospinal fluid). The severity of infection was assessed using Acute Physiology and Chronic Health Evaluation II (APACHE II) score. IL-6 and TNF-α level estimation was done for all patients on day 1, 3, and 7 of hospital stay. The cytokines IL-6 and TNF-α were assessed by sandwich ELISA technique.

### TNF-α estimation

The ELISA plates were coated with monoclonal antihuman TNF-α obtained from mouse. The coating concentration was 4 μg/ml. The reconstituted antibody whose concentration was 720 μg/ml was diluted in phosphate buffered saline (PBS) of pH 7.4 to obtain the required concentration for coating. The coated plates were incubated in room temperature overnight. The coated overnight incubated plates were drained of and then were blocked using block buffer (1% bovine serum albumin (BSA), 5% sucrose in PBS with 0.05% sodium nitrite). The plates were incubated for 2 h at room temperature. The blocking solution descended, and the plates were washed thrice in wash buffer (0.05% Tween 20 in PBS, pH 7.4). The washed plates were then loaded with the standard recombinant human TNF-alpha in different grading concentrations. This is for generalizing a standard curve for the estimation of unknown concentrations in plasma. The standards were run in each plate. (here were 96 wells in each plate of which 16 were taken up by the standards in duplicate.) The remaining wells were loaded with test plasma (undiluted). These plates were incubated for 2 h at room temperature. After 2 h, the plates were again washed as described previously. Biotinylated second antibody was added to the plates, polyclonal antihuman TNF-alpha (source-goat). The reconstituted biotinylated antibody (concentration 54 μg per ml) is diluted to obtain a concentration of 0.3 μg/ml and then used. The plates were incubated for 2 h at room temperature. After 2 h, the plates were washed again. To this is then added the Streptavidin-Horseradish Peroxidise (HRP). The plates were incubated for about 20 min and washed. Tetra methyl benzidine was added and the plates incubated for about 20 min. The coloring reaction was stopped using 2N sulfuric acid and optical density readings taken at 450 nm wavelength Organon Teknika ELISA reader.

### IL-6 estimation

The ELISA plates were coated with monoclonal antihuman IL-6*Ra* obtained from mouse. The coating concentration was 6 μg/ml. The reconstituted antibody whose concentration was 500 μg/ml was diluted in phosphate buffered saline (PBS) of pH 7.4 to obtain the required concentration for coating. The coated plates were incubated at room temperature for 2 h and overnight at 4°C. The coated, overnight-incubated plates were drained off and then were blocked using 4% Bovine Serum Albumin (BSA) solution. The plates were incubated with BSA for 2 h at room temperature. These blocked plates can be sealed and stored at 4°C for subsequent use or can be used right away, the blocking solution (BSA) was discarded and the plates were washed thrice in wash buffer (0.l % Tween 20 in PBS pH 7.4). The washed plates are then loaded with the standard human IL-6Ra in different grading concentrations. This is for generating a standard curve for the estimation of unknown concentrations in the plasma. The standards were run in each plate. (There are 96 wells in each plate of which 16 wells are taken up by the standards in duplicate.) The remaining wells were loaded with test plasma (undiluted). These plates were incubated for 2 h at room temperature. After 2 h, the plates were again washed as described previously.

To the plates are added the biotinylated second antibody, polyclonal antihuman IL-6*Ra* (source rabbit). The reconstituted biotinylated antibody (concentration: 50 μg/ml) is diluted to obtain a concentration of 0.2 μg/ml, and then used. The plates were incubated for 2 h at room temperature. After 2 h, the plates were washed again. Then, the Streptavidin-HorseRadish Peroxidase (HRP) complex was added to this in a dilution of 1:2500. The plates were incubated with the complex for an hour and then washed. Streptavidin HRP complex is followed by coloring substrate Tetra Methyl Benzidine (TMB) directly from ready-to-use solution. After the addition of TMB, the plates were incubated in the dark for about 5-10 min, exact time being decided by a good gradation in color intensity in the standards. The coloring reaction was then stopped using 0.lM H_2_SO_4_, and optical density readings were taken at 450 nm.

### Statistical analysis

Statistical analyses were performed using Statistical Package for Social Survey (SPSS) for Windows 11.0. The main outcome measures in this study were TNF-α and IL-6 on 3 different days. Student and paired ‘t’ tests were applied for comparing different groups among men and women. Correlation analysis was applied to examine the relationship of various variables of interest. Two-way Analysis of Variance (ANOVA) was performed for IL-6 using both males and females as the grouping factor, and later, one-way ANOVA was done for repeated measures as there was a significant difference between the genders. Significant effects on one-way ANOVA were further followed up by post-hoc ‘t’ tests with Bonferroni correction. The values of TNF-α were same on days 3 and 7 and were excluded from the analysis. Two tailed ‘p’ values below 0.05 were considered significant. The results was tabulated and graphically represented using Microsoft Office for Windows 2007.

## Results

**Figure 1:**
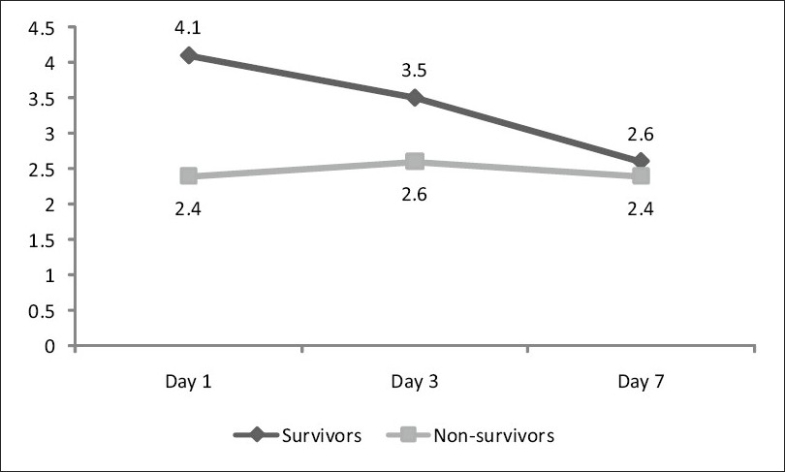
Correlation of IL-6 values on day 1, day 3 and day 7 among survivors and nonsurvivors subgroups of the male subset using the twoway ANOVA test. In survivor group, there is a significant decrease in the level of IL-6 from day 1 to day 7, whereas there is no significant change in the values in the nonsurvivor group

**Figure 2:**
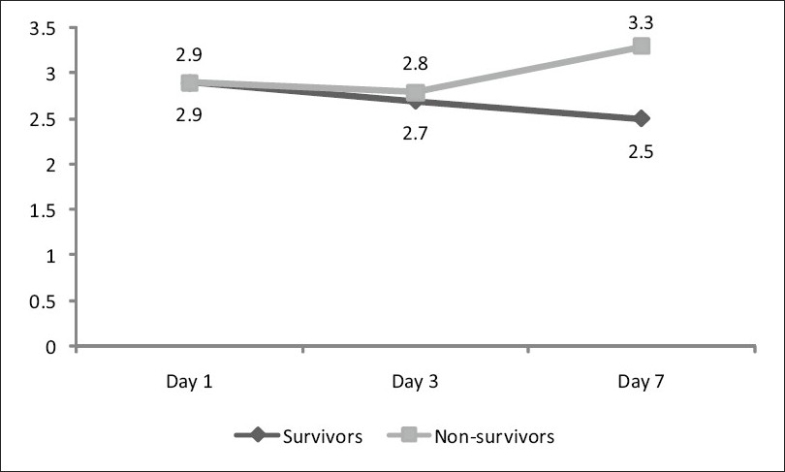
Correlation of IL-6 values on day 1, day 3 and day 7 among survivors and non-survivors subgroups of the female subset using the twoway ANOVA test. In the survivor group, there is a significant decrease in the levels of IL-6 from day 1 to day 7, whereas in the nonsurvivor group, there is an increase in the IL-6 values over the same study period

Of the 30 patients enrolled in the study, 16 were males and 14 females. Respiratory tract infection in the form of pneumonia was the commonest source of infection (12) followed by urinary tract infection (8) and skin infection (3). The source of infection in rest 7 patients' could not be located. Twelve patients with severe sepsis and multi-organ dysfunction succumbed to death 7 days after admission. Four patients (2 male and 2 female) died within 7 days of admission. Two deaths (1 Male and 1 female) were on day 4, and the other 2 were on day 5. The primary cause of death in all these cases was sepsis and multi-organ failure. These cases were not included in day 7 comparison. The results were tabulated on the basis of gender Table 1 and survival Table 2. A correlation of APACHE score, TNF-α values and IL-6 values on day 1, day 3 and day 7 between survivors and nonsurvivors is given in Table 3. IL-6 values on various days in males and females are graphically represented in Figures 1 and 2.

**Table 1:** Distribution of variables among study population (all values in pg/ml) The total number of male and female and ages were well matched. There is no significant difference in the APACHE-II scores of the two groups. The cytokine analysis reveals a significant difference in the TNF-α values on day 1

Variable	Males	Females	Significance
Number	16	14	
Age	70 ± 6	69 ± 5.4	Not significant
APACHE II	23.8 ± 19.1	24.8 ± 16.4	Not significant
TNF-α Day 1	31.8± 22.8	18.1± 15.3	Significant (*P* < 0.00001)
TNF-α Day 3	<10	<10	Not significant
TNF-α Day 7	<10	<10	Not significant
IL-6 Day 1	3.6 ± 1.5	2.9 ± 1.1	Significant (*P* < 0.01)
IL-6 Day 3	3.2 ± 0.7	2.8 ± 1.2	Significant (*P* < 0.02)
IL-6 Day 7	2.6±0.8	3.0±1.2	Significant (*P* < 0.04)

**Table 2:** Correlation of APACHE score, TNF-α values and IL-6 values on day 1, day 3 and day 7 among survivors and non-survivors sub-groups of the male subset (all values in pg/ml). The APACHE-II score was significantly higher in the nonsurvivor group. The IL-6 levels are also significantly different on day 1 and day 3

Variable	Survivors (10)	Nonsurvivors (6)	Significance
Apache-II score	16.2 ± 18.8	39.1 ± 13.7	Significant (*P* < 0.00001)
TNF-α	33.2 ± 17.1	31.9 ± 31.7	Not significant
IL-6 Day 1	4.1 ± 1.5	2.4 ± 0.6	Significant (*P* < 0.00001)
IL-6 Day 3	3.5 ± 0.4	2.6 ± 0.6	Significant (*P* < 0.00001)
IL-6 Day 7	2.6 ± 0.9	2.4 ±0.6	Not significant

**Table 3:** Correlation of APACHE score, TNF-α values and IL-6 values on day 1, day 3 and day 7 among survivors and nonsurvivors subgroups of the female subset. (all values in pg/ml). There is a significant difference in APACHE-II score and TNF-α on day 1 between the survivor and nonsurvivor group

Variable	Survivors (8)	Nonsurvivors (6)	Significance
APACHE-II	11.4 ± 11.2	31.5 ± 14.4	Significant (*P* < 0.00001)
TNF-α	34.2 ± 17.9	10.0 ± 0.02	Significant (*P* < 0.00001)
IL-6 Day 1	2.9 ± 0.8	2.9 ± 1.2	Not significant
IL-6 Day 3	2.7 ± 0.6	2.8 ± 1.4	Not significant
IL-6 Day 7	2.5 ± 0.2	3.3 ± 1.5	Not significant

## Discussion

Cytokine profiling of patients with severe sepsis may be a very useful tool in the prediction of mortality and better management of patients in sepsis.[3,6] A decreasing trend in IL-6 values was associated with a better prognosis in both male and female sub-groups in our study. Conversely, an increasing trend in the pro-inflammatory cytokines was associated with higher mortality. It has been shown in many studies that inflammatory response and cytokine response play a vital role in predicting mortality and prognosis in sepsis in adult patients. Gogos *et al.* concluded from their study that an elevated serum TNF-α level was associated with death, and higher levels of TNF-α and IL-6 were detected in patients with an early hemodynamic deterioration.[8] A significant decrease in the IL-6 level was observed over the study period by Presterl *et al*. in the survivor group only (*P*<0.05).[9] Plotkin from their study for sepsis marker dynamics reported that fast kinetics of C-reactive protein and procalcitonin made it impossible to assess the prognosis and effectiveness of treatment of sepsis using these markers. IL-6, lactoferrin (LF) and sorption ability of erythrocytes (SAE) were the only ones that could be used for this purpose.[10]

The present study showed that pro-inflammatory cytokine release in elderly is similar to other adults, and serial estimation will help in the prediction of sepsis. Similar results have been reported in elderly population in various studies. Marti *et al*., in their study of elderly sepsis patients, found that a higher value of IL-6 on day 4 was associated with higher mortality.[11] Oberholzer *et al.,* in their study of adult sepsis patients to find prognostic markers, concluded that pro-inflammatory cytokine IL-6 was good in augmenting prognostic scores and in predicting outcome in patients with sepsis.[12] Bozza *et al.,* in their multiplex analysis, concluded that that baseline higher values of IL-6 and TNF-α were predictive of higher mortality and organ dysfunction.[13] Kellum *et al.,* in their study of patients with community acquired pneumonia and sepsis, concluded that higher concentrations of IL-6 at admission were predictive of higher mortality.[14]

The mortality in female sepsis patients was higher compared to the male counterpart in the present study. Eachempati *et al*. reported a significantly higher mortality rate in elderly female patients with sepsis.[15] On the contrary, Schroder *et al.* and Adrie *et al.* reported a better outcome among female sepsis patients.[16–17] There are also reports stating no gender-based difference in the outcome of sepsis.[18–22]

Various reasons influence the outcome of sepsis. Sex hormones have long said to influence the outcome in sepsis. Estrogens promote adaptive immune response in sepsis. Social, environmental, economic and personal health-related activities are reported to influence the outcome. There are reports stating lower access to acute health care among females due to cultural phenomena, differential access and socio-economic constraints indirectly influence the poor outcome in females.[23,24]

Low TNF-α value (<10) at the onset in the females was associated with increased mortality in our study (*P* < 0.00001). However no such finding was found in males. This can be explained by the great variation in TNF-α production due to genetic polymorphisms within the TNF loci.[25] Immune response difference between male and female have been studied by a few investigators. Imahara *et al.* found that women produced significantly less TNF-α and IL-1β but not IL-6 in response to exposure to lipopolysaccharide.[26] Moxley *et al.* reported that females have nearly 30% lower innate immune response other than the influence of the HLA-region TNF locus.[27]

## Conclusion

Cytokine profiling for TNF-α and IL-6 level in elderly patients with sepsis helps in prognosticating the outcome. Female gender was an independent predictor of increased morality in critically ill surgical patients with documented infection. However, no studies have been performed on patients with medical illness with sepsis and influence of gender to quality of health care. Further studies are required to corroborate our finding.
